# Bacterial superglue generates a full-length circumsporozoite protein virus-like particle vaccine capable of inducing high and durable antibody responses

**DOI:** 10.1186/s12936-016-1574-1

**Published:** 2016-11-08

**Authors:** Christoph M. Janitzek, Sungwa Matondo, Susan Thrane, Morten A. Nielsen, Reginald Kavishe, Steve B. Mwakalinga, Thor G. Theander, Ali Salanti, Adam F. Sander

**Affiliations:** 1 Centre for Medical Parasitology, Department of Immunology and Microbiology, University of Copenhagen, Copenhagen, Denmark; 2 Department of Infectious Diseases, Copenhagen University Hospital, Copenhagen, Denmark; 3 Kilimanjaro Christian Medical University College (KCMUCo), Moshi, Tanzania

**Keywords:** Virus-like particle, VLP, Pre-erythrocytic, Malaria vaccine, Circumsporozoite protein, CSP, Spycatcher, Spytag, Bacterial superglue, Split-intein

## Abstract

**Background:**

Malaria, caused by *Plasmodium falciparum,* continues to have a devastating impact on global health, emphasizing the great need for a malaria vaccine. The circumsporozoite protein (CSP) is an attractive target for a malaria vaccine, and forms a major component of RTS,S, the most clinically advanced malaria vaccine. The clinical efficacy of RTS,S has been moderate, yet has demonstrated the viability of a CSP-based malaria vaccine. In this study, a vaccine comprised of the full-length CSP antigen presented on a virus-like particle (VLP) is produced using a split-intein conjugation system (SpyTag/SpyCatcher) and the immunogenicity is tested in mice.

**Methods:**

Full-length 3d7 CSP protein was genetically fused at the C-terminus to SpyCatcher. The CSP-SpyCatcher antigen was then covalently attached (via the SpyTag/SpyCatcher interaction) to *Acinetobacter phage* AP205 VLPs which were modified to display one SpyTag per VLP subunit. To evaluate the VLP-display effect, the immunogenicity of the VLP vaccine was tested in mice and compared to a control vaccine containing AP205 VLPs plus unconjugated CSP.

**Results:**

Full-length CSP was conjugated at high density (an average of 112 CSP molecules per VLP) to AP205 SpyTag-VLPs. Vaccination of mice with the CSP Spy-VLP vaccine resulted in significantly increased antibody titres over a course of 7 months as compared to the control group (2.6-fold higher at 7 months after immunization). Furthermore, the CSP Spy-VLP vaccine appears to stimulate production of IgG2a antibodies, which has been linked with a more efficient clearing of intracellular parasite infection.

**Conclusion:**

This study demonstrates that the high-density display of CSP on SpyTag-VLPs, significantly increases the level and quality of the vaccine-induced humoral response, compared to a control vaccine consisting of soluble CSP plus AP205 VLPs. The SpyTag-VLP platform utilized in this study constitutes a versatile and rapid method to develop highly immunogenic vaccines. It might serve as a generic tool for the cost-effective development of effective VLP-vaccines, e.g., against malaria.

## Background

**Fig. 1:**
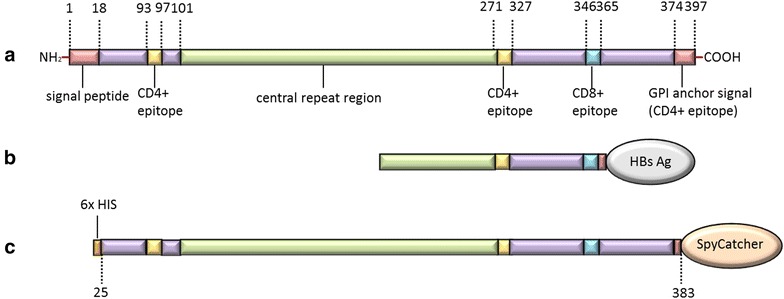
Graphic representation of native *Plasmodium falciparum* CSP compared to RTS,S and the recombinant CSP. **a** Native *P. falciparum* circumsporozoite protein (CSP); **b** Recombinant CSP construct used in the RTS,S vaccine; **c** Recombinant full-length CSP construct used in present study. The full-length CSP sequence (amino acid 25–383) comprising the entire repeat sequence (38 NANP B cell epitopes) as well as T cell epitopes (CD4+ and CD8+) mapped in the N- and C-terminus, was genetically fused at the C-terminus to SpyCatcher

Malaria, caused by *Plasmodium falciparum,* continues to have a devastating impact on global health and is a leading cause of death in children and pregnant women in sub-Saharan Africa [1]. Naturally acquired immunity against malaria is transient and non-sterilizing. There is no commercially available malaria vaccine and the most commonly used interventions are bed nets and drug treatment, which are expensive and progressively ineffective due to drug resistance. The development of a vaccine against malaria has so far been hindered by the sheer complexity of the parasite life cycle [2, 3], antigenic variation [4], as well as an incomplete understanding of the interaction between *P. falciparum* and the human immune system [5]. Vaccines employing radiation-attenuated sporozoites have demonstrated strong and protective immune responses in rodents, primates, as well as human volunteers against malaria infection [6–9], creating a strong rationale for developing vaccines that target the pre-erythrocytic life stage. Due to economic and practical difficulties in relation to developing and implementing a vaccine based on whole sporozoites, the focus has been on developing subunit vaccines using recombinant sporozoite antigens. The circumsporozoite protein (CSP, Fig. 1a) has been identified as the immune-dominant protective antigen in irradiated sporozoites and serves as the antigenic component in the leading malaria subunit vaccine candidate, RTS,S/AS01 (GlaxoSmithKline) [10, 11]. Notably, recent results of a large phase III clinical study showed that the efficacy of the RTS,S/AS01 vaccine was high immediately after the final vaccine dose, but waned quickly in parallel with the decline of vaccine-specific IgG antibody levels. Both in infants (6–12 weeks) and young children (5–15 months) the vaccine efficacy was moderate, 37% in infants [12], and 47% in children [13], when measured over a period of 14 or 12 months, respectively.

The RTS,S vaccine is based on the hepatitis B small surface antigen (HbsAg) particle displaying a truncated CSP antigen at a ratio of 1 CSP-fused HbsAg per 3 unfused HbsAg (Fig. 1b). Emerging data from the hepatitis vaccination programme suggest that the HbsAg particle induces an excellent memory B cell response, yet is ineffective at inducing long-lived plasma cells and sustainable IgG titres, as reviewed in [14]. Hence the RTS,S vaccine faces two major intrinsic obstacles to be an effective pre-erythrocytic vaccine, such as low display of the antigen and a particle backbone that is ineffective in inducing long-lived plasma cells. In addition, the CSP antigen used in the RTS,S vaccine lacks the C-terminal region (i.e. excluding important T cell epitopes) as well as excludes a large portion of the central NANP repeat region (i.e. excluding B cell epitopes). Several other studies have indicated these epitopes are potentially important for induction of a protective immune response [15–17]. However, the partial protective effect of the RTS,S vaccine demonstrates the viability of a CSP-based malaria vaccine and further improvements of the vaccine may improve its clinical efficacy.

There is clear evidence that an ordered, repetitive and dense organization of epitopes, displayed on a rigid surface (e.g. a virus capsid) and not a lipid-based virus-like particle (VLP) (e.g. HbsAg VLPs) leads to induction of strong and durable antibody responses [18, 19]. Recent studies have shown that vaccine antigens presented on VLP platforms effectively induce high titred antibody responses against poorly immunogenic antigens [20, 21]. One of these studies utilizes a split-intein (SpyTag/SpyCatcher) conjugation system to covalently attach antigens on the surface of rigid *Acinetobacter phage* AP205 VLPs. In brief, the AP205 VLPs were modified to present one SpyCatcher, one SpyTag or two SpyTag per VLP subunit. By modifying antigens to include the corresponding binding tag (SpyTag or SpyCatcher) and mixing these with the Spy-VLPs, the antigen is attached to the surface of the rigid VLPs via a covalent bond formed between the SpyTag and the SpyCatcher and is directionally displayed at high density [21]. In the present work, the Spy-VLP platform is used to facilitate directional, high-density display of a full-length CSP construct (Fig. 1c) covalently attached to the surface of AP205 SpyTag-VLPs. To evaluate the VLP-display effect, the immunogenicity of the CSP-SpyCatcher:SpyTag-VLP (CSP Spy-VLP) vaccine was tested against a control vaccine consisting of unconjugated monomeric CSP antigen and untagged AP205 VLPs by immunization of BALB/c mice.

## Methods

### Design, expression and purification of Spy-AP205 VLPs

*Acinetobacter phage* AP205 Spy-VLPs, displaying one SpyTag per VLP subunit, were designed as previously described [21]. In brief, the gene encoding the major AP205 coat protein (Gene ID: 956335) was fused at the N-terminus to the SpyTag peptide sequence (AHIVMVDAYKPTK) along with a flexible linker. The VLP were expressed in *Escherischia coli* One Shot^®^ BL21 Star™ (DE3) cells (Thermo Scientific) cells and purified via an Optiprep™ (Sigma) step (23, 29 and 35%) gradient.

### Design, expression and purification of HIS-CSP-SpyCatcher antigen

The CSP antigen, comprising amino acids 25–383 (i.e. excluding the N-terminal signal peptide and the C-terminal GPI-anchor) of the native 3D7 sequence (Gene ID: 814364), was designed with a hexa-histidine purification tag (HIS) at N-terminus and the SpyCatcher [22] at the C-terminus. A Glycine–Glycine–Serine linker was inserted between the CSP and the SpyCatcher sequence. The HIS-CSP-SpyCatcher gene sequence was finally modified to contain a BamHI and NotI restriction site at the N- and C-terminus, respectively, and was codon-optimized for and expressed in *Trichoplusia ni* insect cells, as described previously [21, 23].

### Electron microscopy

An aliquot of the CSP Spy-VLP vaccine was adsorbed to 200-mesh mica carbon-coated grids and negatively stained with 2% phosphotungstic acid (pH = 7.0). The VLPs were analyzed with an accelerating voltage of 80 kV, using a CM 100 BioTWIN electron microscope (Phillips, Amsterdam). An Olympus Veleta camera was utilized to obtain photographic records and particle sizes were estimated using ImageJ.

### Formulation of CSP Spy-VLP vaccine

**Fig. 2:**
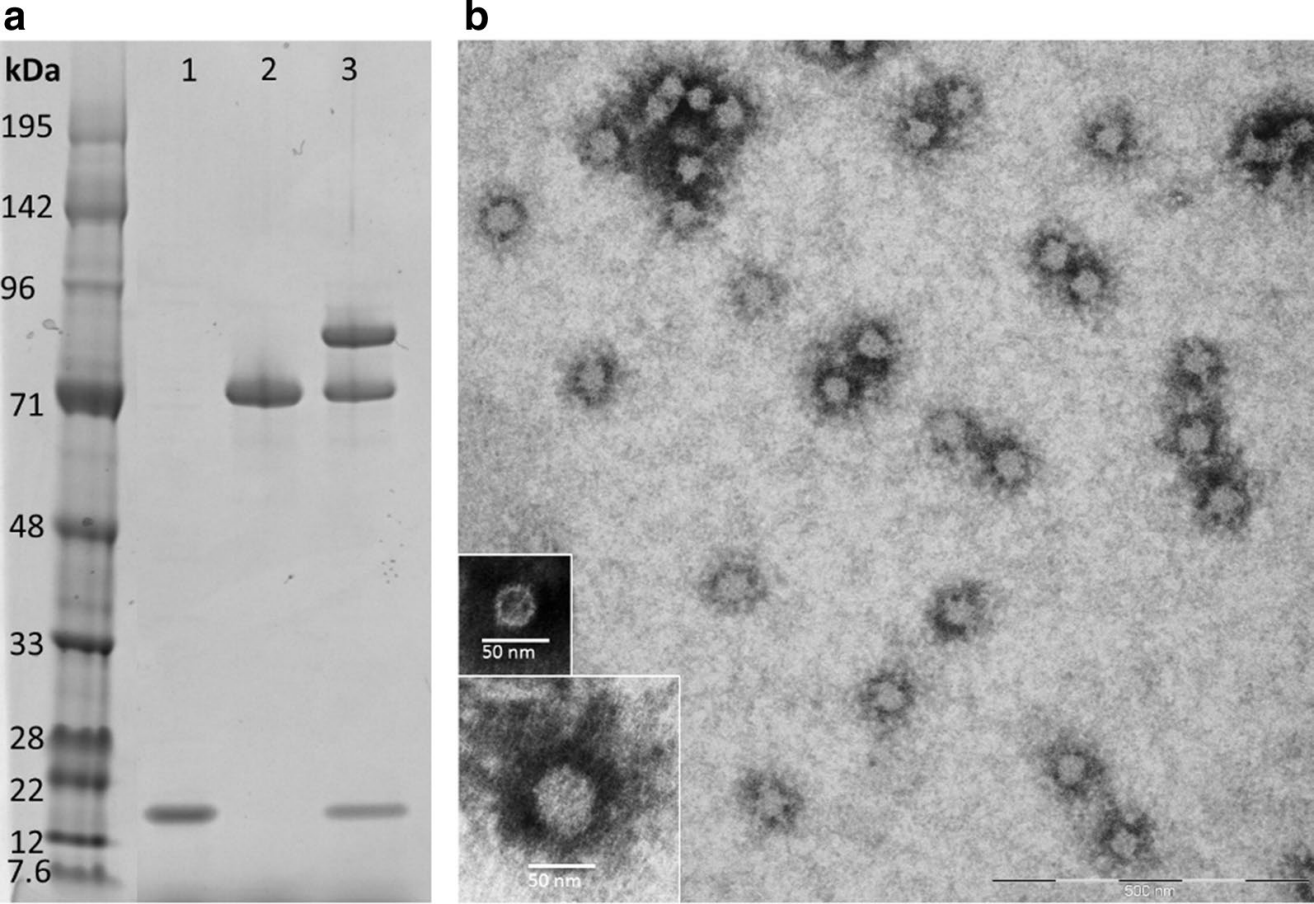
Characterization of a virus-like particle-based CSP vaccine. **a** Reduced SDS-PAGE gels loaded with AP205 SpyTag-VLPs (*lane 1*), CSP-SpyCatcher (*lane 2*) and the CSP Spy-VLP vaccine (*lane 3*), demonstrating that SpyCatcher-CSP has been covaltly conjugated to the AP205 capsid protein. Three protein bands formed following the reaction of SpyTag-VLPs with SpyCatcher-CSP. The *bands* correspond to the size of the CSP-VLP capsid protein conjugate (89 kDa) (*lane 3 top*), uncoupled CSP vaccine antigen (theoretical size 52 kDa—however it appears as 72 kDa on the SDS gel) (*lane 3 middle*) and unconjugated SpyTag-VLP capsid protein (16.5 kDa) (*lane 3 bottom*). Densitometric analysis comparing the protein band intensity of in *lane 1 with the bottom band in lane 3* revealed that an average of 112 CSP-SpyCatcher antigens have been covalently attached per VLP. **b** Transmission electron microscopy of the CSP Spy-VLP vaccine (i.e., CSP-SpyCatcher covalently attached to SpyTag-VLP). CSP Spy-VLP samples were placed on carbon, adsorbed to a grid and negatively stained with 2% phosphotungstic acid. The large image reveals uniformly distributed, non-aggregated particles of approximately 49 nm. The *bottom left images* show a zoomed-in CSP Spy-VLP (*bottom*) compared to an unconjugated AP205 Spy-VLP (*top*)

Purified HIS-CSP-SpyCatcher antigen and AP205 Spy-VLP were mixed at a 1.5:1 (antigen per VLP subunit) molar ratio and incubated overnight at 4 °C in a conjugation buffer (PBS, 0.2% Polysorbate 80, pH 7.2). SDS-PAGE densitometric analysis was used to determine number of VLP monomer subunits that had been conjugated to CSP via the SpyTag/SpyCatcher interaction. Specifically, this amount was assessed by dividing the protein band intensity value of VLP subunit protein band prior to the reaction with antigen (Fig. 2a, lane 1) with the equivalent protein band intensity value after reaction with antigen (Fig. 2a, lane 3, bottom), and multiplied by 100. This percentage was multiplied by 180 (total number of AP205 subunits per VLP). The antigen concentration was adjusted to 5 µg antigen per 100 µl by dilution with PBS buffer (pH 7.2). One hour prior to immunizations, Alhydrogel (2%) (Statens Serum Institut, Copenhagen, Denmark) was added to a final concentration of 2 mg/ml aluminum hydroxide. The control vaccine was prepared in a similar way except using untagged VLPs instead of Spy-VLPs.

### Mouse immunization

Eight-weeks old, female BALB/c mice (Taconic, Denmark) were immunized by intramuscular injection with 100 µl vaccine (corresponding to a dose of 5 μg of CSP per mouse per immunization in both the CSP Spy-VLP vaccine group and the control group) on days 0, 14 and 28. Immune serum was collected on days 14, 28, 42, 148, 192, and 212.

### Antibody response measured by standard ELISA

Measurement of serum immunoglobulin (Ig) levels was done by standard enzyme-linked immunosorbent assay (ELISA) as described previously [21], using 96-well plates (Nunc MaxiSorp) coated with 0.1 µg recombinant CSP protein (no SpyCatcher) per well. The Serum Ig endpoint titres were determined by applying a cut-off of OD490 = 0.2. In addition, the geometric means of the titres were calculated on all measured days and the fold-increase in titres (VLP vaccine group/control vaccine group) was determined. The statistical analysis was performed by a non-parametric, two tailed, Mann–Whitney Rank Sum Test, with statistical significance being defined at threshold P < 0.05.

### Determination of serum IgG subclass profiles

IgG sub-class profiling of anti-CSP mouse sera was performed according to the above described standard ELISA protocol and as described recently [21].

## Results

### Development and characterization of a VLP-based CSP vaccine

The full-length CSP sequence, comprising the entire repeat sequence (38 NANP B cell epitopes) as well as T cell epitopes (CD4+ and CD8+) mapped in the N- and C-terminus, was genetically fused at the C-terminus to the SpyCatcher (Fig. 1c). The fusion construct was expressed in baculovirus transfected insect cells, and 0.5 mg pure (>85%) monomeric antigen was obtained following purification by immobilized metal affinity chromatography and size-exclusion chromatography (Fig. 2a, lane 2).

Mixing and incubation of purified CSP-SpyCatcher antigen with Spy-VLPs was done at a 1.5:1 molar ratio (antigen per VLP subunit), respectively. The Spytag/Spycatcher conjugation should result in exhaustion of both protein components in the reaction [24]. The fact that there was a residual, non-conjugated Spy-VLP band on the SDS-page gel (Fig. 2a, lane 3, bottom) indicated that steric hindrance may prevent any further conjugation of CSP on the fully, densely covered particle, causing not all VLP subunits to be conjugated with an antigen. The densitometric analysis revealed that there was an average of 112 CSP antigens per VLP (Fig. 2a). The final vaccine did contain excess non-conjugated CSP, as it was deemed that this, if anything, would only pose a negative contribution to the immune response and would thus not introduce a bias favouring the VLP display effect (Fig. 2a, lane 3).

Transmission electron microscopy revealed a homogenous population of non-aggregated antigen-VLP complexes with an estimated size of 49 nm (Fig. 2b). The estimated diameter of the CSP Spy-VLP, moreover, appears bigger compared to unconjugated Spy-VLP, with an estimated size of 30 nm (Fig. 2b, bottom left images), indicating the presence of conjugated antigen on the surface of the Spy-VLPs.

### Immunogenicity of the CSP Spy-VLP vaccine

**Fig. 3:**
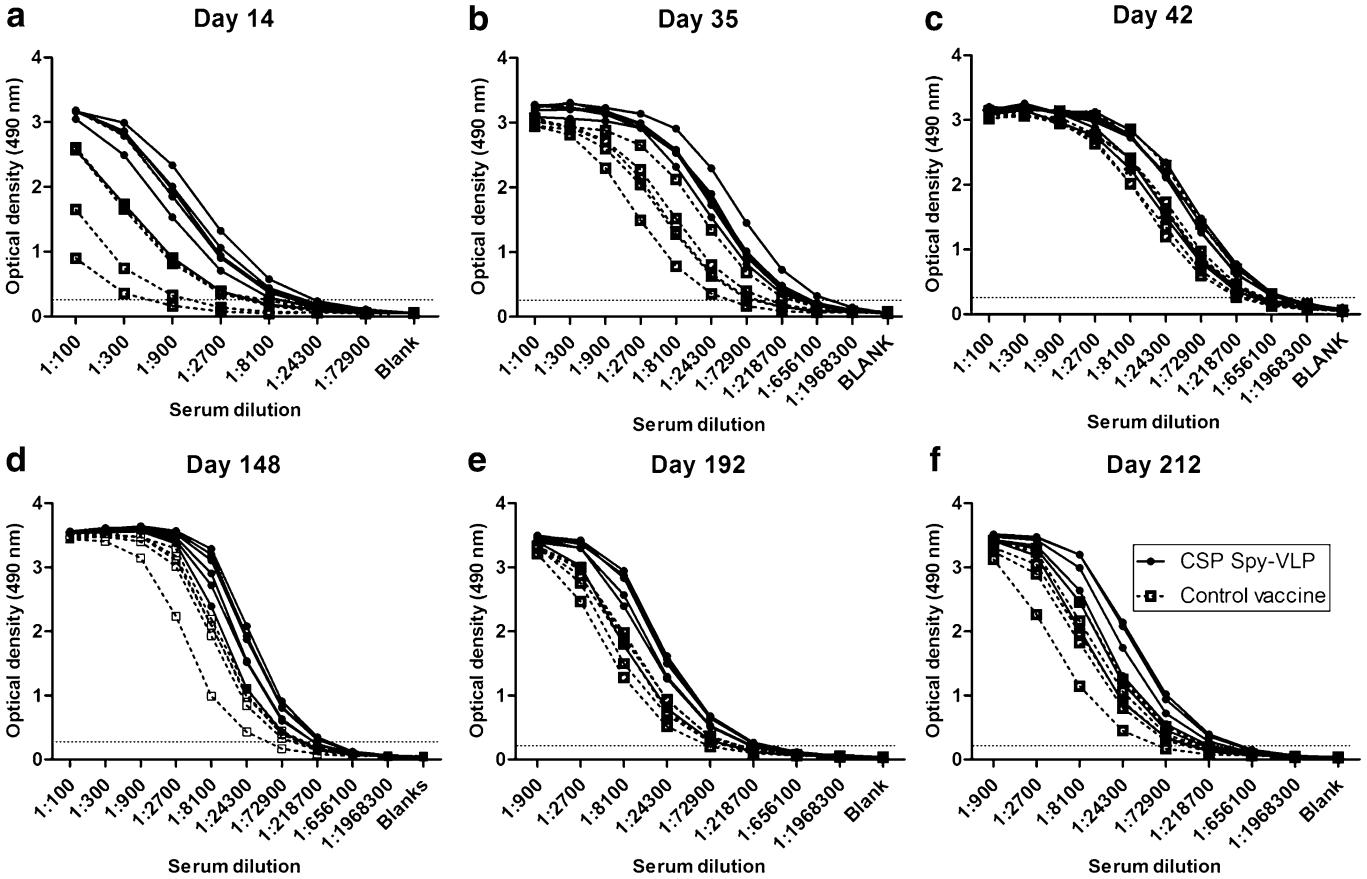
CSP-specific IgG levels in mice after immunization with soluble or Spy-VLP displayed CSP antigen at **a** day 14, **b** day 35, **c **day 42, **d** day 148, **e** day 192 and **f** day 212 after first immunization. CSP-specific IgG levels (OD ELISA) in serum from mice (n = 6 CSP Spy-VLP group, n = 5 control vaccine group) immunized with a CSP Spy-VLP vaccine (*filled circles*) or with a control vaccine consisting of soluble CSP mixed with untagged AP205 VLPs (*open squares*). Both vaccines were formulated using aluminum hydroxide adjuvant. Mice were immunized on days 0, 14 and 42. Differences in titres between vaccination groups were analysed using OD cut-off 0.2 and Mann–Whitney Rank Sum test; day 14 (P = 0.0169), day 28 (P = 0.0109), day 42 (P = 0.0578), day 148 (P = 0.0116), days 192 and 212 (P = 0.0316)

The immunogenicity of the Spy-VLP based CSP vaccine, formulated with aluminum hydroxide adjuvant, was assessed by evaluation of vaccine-induced humoral responses in mice. Specifically, BALB/c mice were immunized intramuscularly on days 0, 14 and 28 and anti-CSP Ig titres were subsequently measured by ELISA in sera obtained at days 14, 28, 42, 148, 192, and 212 (Fig. 3). At days 14 and 28 anti-CSP Ig titres were significantly higher in mice receiving the CSP Spy-VLP vaccine compared to mice immunized with the unconjugated control vaccine (P < 0.02). At day 42 there was no statistically significant difference (P = 0.058) in the level of anti-CSP Ig between the two groups. However, measurement of anti-CSP Ig titres at days 148, 192 and 212 again showed significantly higher anti-CSP Ig titres in CSP -VLP immunized mice (P = 0.01, day 148 and P = 0.03, days 192 and 212).

**Fig. 4:**
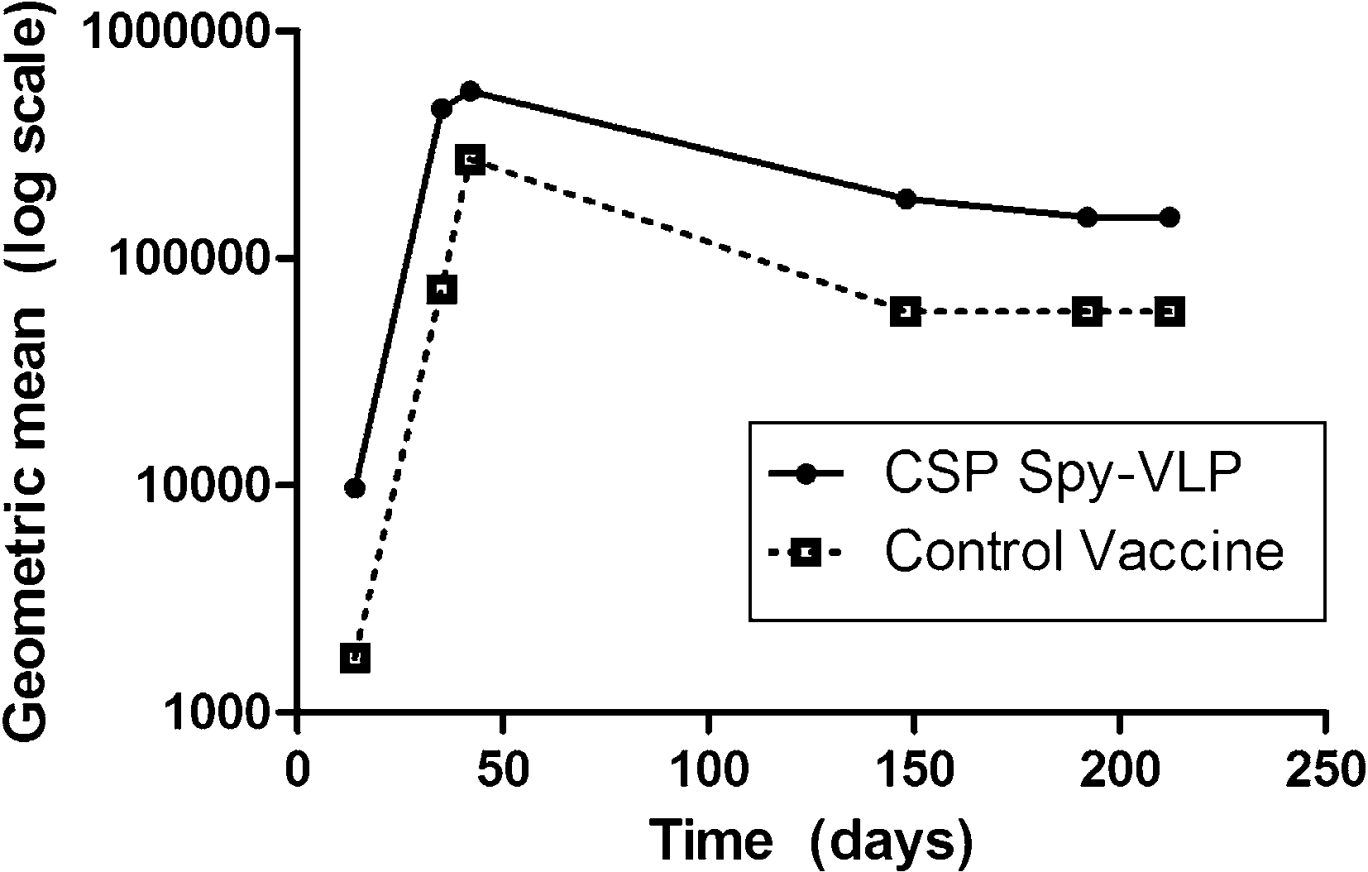
Development of geometric mean titres over time. The geometric mean titres were calculated for the CSP Spy-VLP vaccine group (*filled circles*) and the control vaccine group (*open squares*) at measured days (14, 28, 42, 148, 192, and 212) and plotted on a log-scale. Both immunization groups appear to reach a plateau from day 148 and onward. The titre plateau of the CSP Spy-VLP immunized group is 2.6-fold increase higher compared to the titre of the control vaccine group at days 192 and 212

Throughout this study, the geometric means of the CSP Spy-VLP vaccine group were higher compared to the control vaccine-immunized group (Fig. 4). This corresponds to a 5.6-, 6.2-, 2-, 3-, 2.6-, and 2.6-fold increase in geometric mean titres in the CSP Spy-VLP vaccine group on days 14, 28, 42, 148, 192, and 212, respectively. At day 148 and forth the geometric mean titre appeared to reach a plateau in both immunization groups.

### Subclass profiling of vaccine-induced IgG

**Fig. 5:**
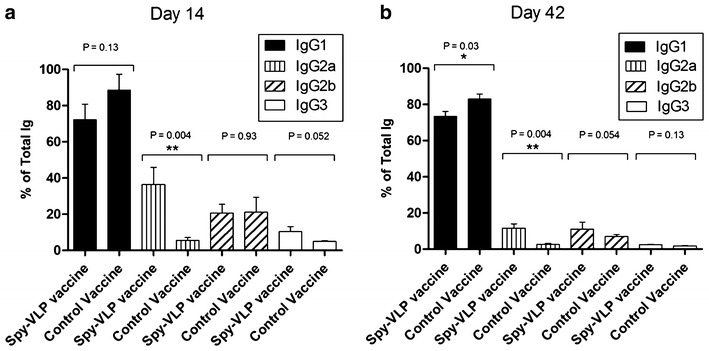
Anti-CSP IgG subclass profiling performed at **a** 14 days and **b** 42 days after first immunization. The proportion of anti-CSP IgG1, IgG2a, IgG2b and IgG3 in sera, obtained at day 14 and 42, relative to the total vaccine-induced IgG response in vaccinated mice (n = 6 for CSP-VLP vaccinated group and n = 5 for soluble CSP vaccine group). Mann–Whitney Rank Sum test was used for statistical comparisons

The relative proportion of IgG subclasses elicited in mice immunized with the CSP Spy-VLP vaccine and the unconjugated control vaccine were compared, as previously described [21]. Measurements were performed on sera obtained at days 14 and 42 (Fig. 5). Whereas IgG1 was the dominant IgG subclass in all analysed sera there were a significantly increased proportion of anti-CSP IgG2a antibodies in mice immunized with the CSP Spy-VLP vaccine compared to the control group. At day 42 the VLP-immunized group showed a (not statistical significant) tendency to produce more IgG2b. The IgG3 subclass was nearly absent in all analysed sera.

## Discussion

The ultimate goal of active vaccination is to induce prolonged, preferably life-long, protective immune responses, and the main effector mechanism of most prophylactic vaccines is the generation of protective antibodies [25]. Humoral memory depends on two layers of defence: (1) memory B cells that are able to rapidly reactivate to produce antibodies upon antigen exposure; and, (2) pre-existing protective antibodies secreted by long-lived plasma cells [26]. In the context of designing a malaria vaccine to prevent liver infection by the transient sporozoite form of the parasite, sufficient titres of circulating antibodies to protect from disease are likely required [27]. However, successful induction and persistence of long-lived plasma cells remains a chief challenge in modern vaccine development and has generally not been achieved using simple subunit vaccines [28]. Remarkably, the licensed Human Papillomavirus (HPV) vaccines are able to induce stable antibody titres after a single immunization [19], which has been ascribed to the ordered, repetitive and dense display of surface epitopes offered by the rigid icosahedral L1 VLPs. In contrast, the hepatitis B vaccine lacks the ability to induce durable antibody titres, even after a prime-boost-boost regimen and protection seems, in this case, to depend on memory B cells rather than on long-lived plasma cells [14, 29, 30]. The HBV vaccine is composed of the HBsAg, presented, at low density (100–120 HBsAg per VLP), on the surface of non-rigid lipid particles [31, 32], and it has been proposed that HBV particles represent a sub-optimal virus-like epitope display compared to HPV L1 VLPs [33]. Accordingly, since it appears that the RTS,S vaccine is protective only while antibodies are circulating, HBsAg VLPs (i.e., the hepatitis B vaccine) are likely a sub-optimal platform for displaying the CSP antigen. On that basis it seemed plausible that a vaccine presenting the full-length CSP at high density on the surface of a rigid particle platform (i.e., resembling the HPV L1 VLP epitope display) would enhance the quality, magnitude and breadth of protective antibody and T-cell responses. To this end, a recent study reported on the development of a Spy-VLP display platform, enabling uni-directional presentation of large antigens at high density on the surface of rigid AP205 bacteriophage VLPs, and demonstrated the capacity of the platform to induce high-titred, functional, anti-malaria antibodies [21].

The full-length CSP-SpyCatcher antigen construct expressed well in baculovirus transfected insect cells and was compatible with the SpyTag/SpyCatcher VLP conjugation system, i.e., antigen:VLP complexes did not tend to aggregate and precipitate as sometimes occurs during the SpyTag/SpyCatcher-mediated, antigen-VLP conjugation reaction [21]. The estimated number of antigens required to cover the VLP surface matched what would be expected based on the size of the CSP-SpyCatcher protein (53 kDa) [21]. Compared to previously tested CSP-based VLP vaccines, the average antigen display capacity of the CSP Spy-VLPs is three to four times higher [34]. This may constitute a crucial difference as epitope density is an important factor for B cell activation [35, 36].

By testing the CSP Spy-VLP vaccine against a control vaccine containing SpyCatcher-CSP antigen and untagged AP205 VLPs it was possible to directly measure the VLP display-effect. Measurement of vaccine-induced IgG in sera from immunized mice showed that the VLP display gave rise to a significantly higher anti-CSP IgG titres after the first two immunizations, which was again the case after 5–6 months. When analysing the geometric mean titres over time, both in the CSP Spy-VLP-immunized group as well as the control group, the antibody titres plateau after 5–6 months. However, the level of the CSP Spy-VLP-induced antibody titre plateau is moderately, yet significantly higher (2.6-fold increase) compared to the control group. These results indicate that the Spy-VLP display of the CSP antigen trigger a more efficient induction of long-lived plasma cells.

Production of AP205 VLPs in *E. coli* result in encapsidation of host cell RNA, which can pose an adjuvant effect via TLR 7 and 8. Besides, contaminating endotoxin was not removed from the VLP preparations. It is thus feasible that bacterial RNA and/or endotoxins have affected the humoral responses induced by the Spy-VLP and unconjugated control vaccine which could partly explain why the control vaccine, albeit to a lower degree, also managed to induce a long-lasting response.The overall IgG isotype profile showed a predominant induction of IgG1 in both test groups, although there was a statistically significant increase in the level of induced anti-CSP IgG of the 2a subclass in VLP-immunized mice at both measured days. Whether this finding possesses any biological significance remains to be tested. However, the IgG2a subclass has previously been linked with more efficient clearing of intracellular parasites [37, 38] and anti-CSP IgG2a antibodies have been associated with protection against transgenic sporozoite challenge in mice [39].

The antigen used in the RTS,S vaccine is an N-terminally truncated CSP sequence fused to the HBsAg and co-expressed with unfused HBsAg in yeast cells [40, 41]. Since the N-terminal domain of CSP represents a target of protective immunity [16] the N-terminally truncated antigen design may not be optimal [42]. In this study the novel Spy-VLP platform was employed to facilitate virus-like display of a full-length recombinant CSP antigen, which in contrast to previous multivalent displayed CSP constructs [34, 43–45] contains all 38 NANP repeat epitopes of the native sequence as well as important T- and B-cell epitopes mapped to the N-terminal region [46–48].

## Conclusion

The CSP Spy-VLP vaccine appears to improve the response against the CSP protein measured on parameters potentially important for protection. The high-density directional display of full-length CSP on the surface of rigid AP205 Spy-VLPs resulted in significantly increased levels of anti-CSP antibody responses in vaccinated mice over the course of 7 months. Furthermore, the VLP-display seemed to cause an increased induction of IgG2a antibodies, which have been associated with efficient clearing of intracellular parasite infection. Studies to compare this vaccine to RTS,S head-to-head, employing extrinsic adjuvants, may help decide on further clinical development. Finally, this study further supports the use of the Spy-VLP system as a versatile, cost-effective, and rapid method to develop highly immunogenic vaccines e.g. against malaria.

## Abbreviations

CSP: cirucumsporozoite protein
CSP Spy-VLP: CSP-SpyCatcher:SpyTag-VLP
ELISA: enzyme-linked immunosorbent assay
HbsAg: hepatitis B small surface antigen
HIS: hexa-histidine purification tag
HRP: horseradish peroxidase
Ig: immunoglobulin
PBS: phosphate-buffered saline
Sf9: *Spodoptera frugiperda*
RT: room temperature
VLP: virus-like particle
